# The *M. tuberculosis* Phosphate-Binding Lipoproteins PstS1 and PstS3 Induce Th1 and Th17 Responses That Are Not Associated with Protection against *M. tuberculosis* Infection

**DOI:** 10.1155/2011/690328

**Published:** 2011-03-27

**Authors:** Carla Palma, Ralf Spallek, Giovanni Piccaro, Manuela Pardini, Fatima Jonas, Wulf Oehlmann, Mahavir Singh, Antonio Cassone

**Affiliations:** ^1^Department of Infectious, Parasitic and Immune-Mediated Diseases, Istituto Superiore di Sanità, Viale Regina Elena 299, 00161 Rome, Italy; ^2^Lionex Diagnostic and Therapeutics GmbH, Salzdahlumer Strasse 196, 38126 Braunschweig, Germany

## Abstract

The *M. tuberculosis* phosphate-binding transporter lipoproteins PstS1 and PstS3 were good immunogens inducing CD8^+^ T-cell activation and both Th1 and Th17 immunity in mice. However, this antigen-specific immunity, even when amplified by administration of the protein with the adjuvant LTK63 or by the DNA priming/protein boosting regimen, was not able to contain *M. tuberculosis* replication in the lungs of infected mice. The lack of protection might be ascribed with the scarce/absent capacity of PstS1/PstS3 antigens to modulate the IFN-*γ* response elicited by *M. tuberculosis* infection during which, however, PstS1-specific IL-17 secreting cells were generated in both unvaccinated and BCG-vaccinated mice. In spite of a lack of protection by PstS1/PstS3 immunizations, our results do show that PstS1 is able to induce IL-17 response upon *M. tuberculosis* infection which is of interest in the study of anti-*M. tuberculosis* immunity and as potential immunomodulator in combined vaccines.

## 1. Introduction

Tuberculosis (TB) remains a leading human infectious disease and a major public health problem in low-income countries [1]. Despite the availability of the Bacillus Calmette-Guérin (BCG) vaccine for more than 80 years, until now an effective tuberculosis vaccine is still unavailable and still unknown are the correlates of protection against this disease [2]. Several protective antigens in particular those belonging to the immunodominant complex Ag85 [3–5] have been studied but immune responses against most of them are still not precisely defined. Hence, the search for new protective *Mycobacterium tuberculosis (M. tuberculosis)* antigens remain a vital field, particularly for proteins exposed on the bacterial cell surface exerting important physiological functions and/or acting as virulence factors. 

We recently focused our attention on phosphate-specific transporter (pst) lipoproteins of *M. tuberculosis*. Pst is a membrane-associated complex that belongs to the ABC transporter superfamily [6]. In *M. tuberculosis*, three putative pst operons have been identified pstS1, pstS2, and pstS3 [7], which probably constitute a subtle biochemical adaptation of this microorganism for its growth and survival under different phosphate-limiting conditions during its infectious cycle [8]. The three genes coding these proteins are very similar (about 75% similarity between *pstS1* and *pstS2* or *pstS3* and 94% similarity between PstS2 and PstS3), and all proteins have a lipoprotein consensus signal [8]. These phosphate transport receptors are exposed not only on the cell surface of *M. tuberculosis* but also on the surface of *M. bovis* BCG [8].

Disruption of genes encoding *pstS1* and *pstS2* reduced *in vivo* multiplication of *M. tuberculosis* suggesting that the high-affinity phosphate-specific transporters are also virulence factors of *M. tuberculosis* and *M. bovis* [9]. Specific immunity against PstS lipoprotein has been detected in TB patients, and in particular antibodies (Abs) against PstS1 have been reported to be a valuable tool in the serodiagnosis of active TB [10–12]. Moreover, mice vaccinated with DNA coding for *pstS3* demonstrated significant and sustained reduction in bacterial load in spleen and lungs for 3 months after *M. tuberculosis* challenge, as compared with CFU counts in control unvaccinated mice [13]. Conversely, immunizations with DNA coding for *pstS1* resulted in contradictory evidence regarding protection [13, 14] possibly due to different plasmid vector backbone and different experimental protocol of immunization and infection.

In this paper, we have reassessed the role of PstS3 and PstS1 antigens on B and T cell-mediated immunity and protection against *M. tuberculosis* infection. To expand and diversify the immune responses induced by immunizations with DNA only, we used a combination of DNA and protein in a prime-boosting regimen and also the protein administered with the adjuvant LTK63. In fact, our previous studies on Ag85B, an abundant secreted protein of replicating MTB which is currently evaluated in various TB vaccine formulations [5], revealed that antigen-specific immunity can be enhanced by priming/boosting regimen [3]. In addition, the potent mucosal as well as systemic adjuvant LTK63, a nontoxic derivative of heat-labile enterotoxin of *Escherichia coli* [15], can exert positive and negative control on Ag85B-specific Th1 polarization [4, 5]. The attention was focused especially on the Th1 and Th17 responses, in view of their relevant role in TB immunity. The importance of IFN-*γ*-producing CD4^+^ T cells in primary resistance to *M. tuberculosis* has been established both in humans and mice [16–18]. IL-17, a proinflammatory cytokine mostly produced by Th17 cells, can provide IFN-*γ*-dependent or IFN-*γ*-independent protection to M. tuberculosis infection [19, 20]. On the other hand, both the IFN-*γ*- and/or IL-17-induced inflammation needs to be tightly controlled during *M. tuberculosis* infection otherwise they can have important pathological consequences [20–24]. 

Therefore the aim of the work was to dissect the PstS1- and PstS3-specific immunity, through the use of different protocols of immunizations, suitable to the identification of protective responses. Some of the immune responses induced by PstS1 and PstS3 have been compared with those induced by Ag85B, taken as protective antigen.

## 2. Materials and Methods

### 2.1. Ethics Statement

The handling of mice was conducted in accordance with the regulations set forward by the institutional animal care committee of the Italian Ministry of Health, and in compliance with European Community Directive 86/609 and the US Association for Laboratory Animal Care recommendations for the care and use of laboratory animals.

### 2.2. Microorganisms

*M. tuberculosis* H37Rv (ATCC 27294) and *M. bovis* BCG strain Pasteur (ATCC 27291) were grown at 37°C in Middlebrook 7H9 medium supplemented with albumin-dextrose-catalase enrichment under agitation (120 rpm) up to mid-exponential phase. Aliquoted stocks were stored at −70°C until use; the titers of stocks were verified on a regular basis by counting the numbers of colony forming unit (CFU) on Middlebrook 7H10 agar plates. For the manipulation of plasmids, *Escherichia coli (E. coli) *(strains: MC1061, K12 CAG629, and Rosetta) was grown on Luria-Bertani (LB) broth or agar supplemented with ampicillin (100 *μ*g/mL), as required.

### 2.3. Production of DNA-PstS1, DNA-PstS3, or DNA-Ag85B Vaccines and Recombinant PstS1, PstS3, or Ag85B Proteins

Plasmid DNA coding for PstS1 or PstS3 was prepared as previously described [13, 14, 25]. Briefly, plasmid DNA was isolated from *E. coli* host strains by standard plasmid preparation technique using the QIAGEN Plasmid Maxi Kit according to the manufacturers recommendations. Bacteria were cultured over night in LB Broth containing the appropriate antibiotics. Following plasmid isolation, the quality of DNA was determined using agarose gel electrophoresis. All plasmid DNA contained their own signal sequence and the human tpa signal sequence. The plasmid sequences were verified by DNA sequencing. Recombinant PstS1 protein was prepared in *E. coli* strain K12 CAG629 containing the expression plasmid for recombinant PstS1 [25]. After cell disruption by sonication, the inclusion body bound protein was solubilized in buffer containing 8 M urea and refolded by gel filtration on Sephadex G25 fine medium (GE Healthcare). Protein contaminants were separated from PstS1 by performing anion exchange chromatography using Q Sepharose High Performance (GE Healthcare). The protein solution was concentrated by crossflow filtration with a module of 100 kDa molecular weight cutoff to a concentration of approximately 1 mg/mL. Finally a buffer exchange step into volatile buffer was performed by gel filtration on Sephadex G25 fine medium (GE Healthcare), and the protein was freeze dried. Recombinant PstS3 protein was prepared in *E. coli* strain Rosetta (DE3) (Novagen) containing the expression plasmid for recombinant PstS3. Cells were grown in LB medium in shaking flasks at 37°C and induced with 1 mM IPTG. 4 h after induction cells were harvested. After cell disruption by sonication the inclusion body bound protein was solubilized in buffer containing 8 M urea. A metal chelate affinity chromatography under denturating conditions was performed (Ni-NTA resin, QIAGEN), and bound protein was eluted in an increasing imidazole gradient. PstS3 containing fractions were refolded by gel filtration on Sephadex G25 fine medium (GE Healthcare) in potassium phosphate buffer. The refolded protein was concentrated using VivaCell concentrators (10 kDa molecular weight cutoff; Sartorius stedim) to a concentration of approximately 150 *μ*g/mL and stored at 4–8°C. The LPS content of all protein preparations was measured by a *Lymulus* amebocyte lysate test and shown to be below 4.3 EU/*μ*g of protein. 

Recombinant Ag85B protein and plasmid DNA coding for Ag85B were prepared as previously described in [3, 4].

### 2.4. Immunization and Mycobacterial Infection

**Figure 1:**
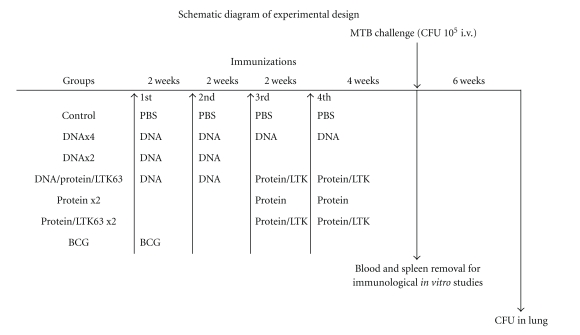
Schematic diagram of experimental design. C57BL/6 female mice were immunized twice or four times at 2-week intervals, with plasmid DNA (coding for *pstS1*, *pstS3,* or *ag85B*) or recombinant proteins (PstS1, PstS3, or Ag85B) in the presence or absence of LTK63 adjuvant. As a positive control, a single dose of BCG was injected s.c. Four weeks after the last immunization, mice were challenged i.v. with 10^5^ CFU of *M. tuberculosis *H37Rv or killed to recover blood and spleen. Control: naive C57BL/6 female mice receiving PBS; DNA: plasmid DNA coding *pstS1*, *pstS3,* or *ag85B* 50 *μ*g/injection i.m.; protein: PstS1, PstS3, or Ag85B proteins 10 *μ*g/injection s.c.; protein/LTK63: PstS1, PstS3, or Ag85B protein together with the adjuvant LTK63 10 *μ*g each/injection s.c.; BCG: BCG Pasteur 10^5^ CFU s.c.

C57BL/6 female mice were supplied as specific pathogen-free mice by Harlan (Udine, Italy) and were maintained in specific pathogen-free conditions. Food and water were available ad libitum. Seven- to 8-week-old mice were immunized. Fifty *μ*g of plasmid DNA-*pstS1*, DNA-*pstS3*, or DNA-*Ag85B* was injected i.m. in 50 *μ*l PBS into the hind leg. On the dorsum of the mice, 10 *μ*g of recombinant PstS1, PstS3, or Ag85B protein was administered s.c with or without 10 *μ*g LTK63 (Novartis Vaccine and Diagnostics Srl, Siena, Italy). Mice were immunized, at 2-week intervals, 2 or 4 times with DNA, and 2 times with recombinant protein alone or together with LTK63 adjuvant. Some mice were boosted twice with recombinant Ag85B protein coadministered with LTK63 adjuvant after priming with DNA. As a positive control, a single dose of BCG (10^5^ CFU) was injected s.c. (Figure 1).

Four weeks after the last immunization, mice were challenged i.v. in a lateral tail vein with 10^5^ CFU of *M. tuberculosis *H37Rv. Infection studies were performed in a biosafety level 3 facility; mice were housed in microisolator cages and fed with autoclaved food and water at libitum. After 4 weeks, the mice were killed by cervical dislocation, and the number of bacteria in lungs was enumerated by homogenizing the tissue and plating 10-fold dilutions, prepared in distilled water, on Middlebrook 7H10 agar. The colonies were counted visually after 21 days of incubation.

### 2.5. Antibody Measurement

Sera from immunized mice were collected by retro-orbital bleeding 4 weeks after the last immunization. The levels of total anti-PstS1 and anti-PstS3 IgG antibodies (Abs) were determined by ELISA. Briefly, polyvinyl microtiter plates (Nunc) were coated overnight at 4°C with 10 *μ*g/mL each recombinant antigen (PstS-1, PstS-3) in PBS. Several dilutions of mouse serum were incubated for 45 min at 37°C prior to the addition of antimouse immunoglobulin G-peroxidase conjugate and then tetramethylbenzidine substrate. The mean absorbance of naive mouse sera, diluted 1 : 100, plus 3 standard deviations was adopted as the cutoff absorbance for determining Ab titers.

### 2.6. Splenocyte Preparation and Cell Culture

Four weeks after the last immunization, single cell suspensions were prepared from the pooled spleens (3 mice/group), passed through Falcon 2360 cell strainers (BD Discovery Labware), centrifuged, aliquoted, and then frozen in liquid nitrogen. Some spleen cells were prepared from unvaccinated mice or BCG-vaccinated mice infected with *M. tuberculosis* as described above. Spleens of infected mice were recovered after 4 or 11 weeks from the challenge and processed in a biosafety level 3 facility. From single spleen cell suspensions, depletion of CD4^+^ T-cell population was obtained by the magnetically labeled fractions isolated from the specific mouse CD4^+^T-cell isolation kits (Miltenyi Biotec Inc., Auburn, CA), in accordance with the manufacturer's instructions. By FACS analysis, negligible fluorescence was observed in depleted fractions during the labeling of cells with the monoclonal Ab CD4-PE corresponding to the depleted cell population. Cells isolated from spleens of naïve, vaccinated, or MTB-infected mice were cultured at 4 × 10^5^/200 *μ*l in 96-well flat plates in RPMI-1640 supplemented with 10% heat-inactivated FBS, 2 mM L-glutamine, 10 mM HEPES buffer, 50 *μ*M 2-*β*-Mercaptoethanol, 50 U/mL penicillin, and 50 *μ*g/mL streptomycin (complete RPMI, cRPMI) and stimulated with Ag85B, PstS1, or PstS3 proteins (5 *μ*g/mL each).

### 2.7. Cytokine Detection

Culture supernatants after 4 days of culture were assayed for IFN-*γ* and IL-17, by specific quantitative sandwich ELISA Kits (mouse Quantikine, R&D System, Inc., Minneapolis, MN), in accordance with the manufacture's instructions. Quantitation was made against a standard curve obtained for individual cytokine standards provided by the manufacturer.

### 2.8. Cell Proliferation by CFSE Staining

Spleen cells (10^7^ cells/mL) were stained with 5(6)-Carboxyfluorescein diacetate N-succinimidyl ester (CFSE) (Invitrogen Life Technologies) at 1 *μ*M in PBS 1% FBS for 10 min at 37°C in the dark. Cells were washed and cultured in 96-well plates, as previously described, for 4 days. After the incubation time, cells were washed with FACS buffer and stained for 20 min at 4°C with PE antimouse CD4 and PerCP antimouse CD8, (BD Biosciences Pharmingen) or the isotype controls. Cells were washed and analyzed on a FACScan flow cytometer with the Cell Quest software programs.

### 2.9. Cell Proliferation by H^3^-Thymidine Incorporation

After 4 days of culture, the cells were pulsed with [H^3^]thymidine (1 *μ*Ci/well) (Perkin Elmer Life and Analytical Sciences, Boston, MA) for additional 18 h. Incorporation of [H^3^]thymidine was measured by *β*-scintillation counting (Micro*β* counter, Perkin Elmer). Values were expressed as mean counts per minute (cpm) in the cultures.

## 3. Results

### 3.1. Immunization with Protein Was Required to Induce Specific Anti-PstS1 or Anti-PstS3 Ab Responses, with PstS1 Being a Better Inducer

In a first series of experiments, mice were immunized with the antigens PstS1 and PstS3 by using several protocols of immunizations, as reported in Figure 1. In particular, DNA was given two or four times, protein, alone or together with LTK63 adjuvant, two times, and priming with two DNA injections and boosting with the protein in LTK63 adjuvant twice. These protocols were selected based on previous work made with the immunodominant *M. tuberculosis* antigen Ag85B, which generated differential humoral and cell-mediated responses associated or not with protection against *M. tuberculosis* challenge (Table 1). 

**Figure 2:**
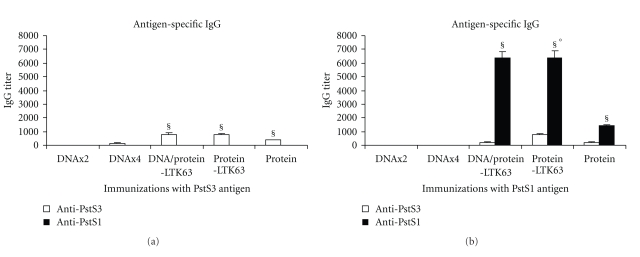
PstS1 and PstS3-specific Ab production in immunized mice. Pooled serum samples (3 mice/group) of mice immunized with antigen PstS3 (a) or PstS1 (b) were analyzed by ELISA for the presence of anti-PstS1 or anti-PstS3 Abs using a conjugated secondary Abs specific total IgG. Data, combined from 3 independent experiments, are plotted as geometric mean ELISA titer ± SEM. ^§^Statistical significant difference among anti-PstS1 Ab titer and anti-PstS3 Ab titer in each group of immunized mice (*P* < .05 or *P* < .01 determined by a two-tailed Student's *t*-test); °statistical significant difference in anti-PstS1 Ab titer between mice immunized with PstS1 protein alone and mice immunized with protein together with LTK63 adjuvant, *P* < .01 determined by a two-tailed Student's *t*-test.

Four weeks after the last immunization, the sera were analyzed for specific anti-PstS1 or anti-PstS3 immunoglobulins (Ig) (Figures 2(a) and 2(b)). Specific IgG were detected exclusively in mice receiving protein with or without adjuvant. PstS1 antigen was a good inducer of antigen-specific Ab response while the Ab-specific production in mice immunized with PstS3 was very weak. In addition, in mice immunized with PstS1 the adjuvant LTK63 greatly enhanced the anti-PstS1 Ab production, and a cross-reactivity with PstS3 antigen was also observed. On the other hand, in mice immunized with PstS3 antigen the antigen-specific IgG titer was minimally enhanced by the adjuvant LTK63 and no cross-reactivity with PstS1 antigen was found. These data suggested a superior capacity of PstS1 to act as cross-stimulating Ab inducer.

### 3.2. Antigen-Specific Cell Proliferation Was Mainly due to CD8^+^ T Cells and Was Higher in PstS1- than in PstS3-Immunized Mice: PstS1 Fully Activated Cells of Mice Immunized with PstS3 Antigen while the Effect of PstS3 Re-Stimulation Was Only Partial on Cells of PstS1-Immunized Mice

**Figure 3:**
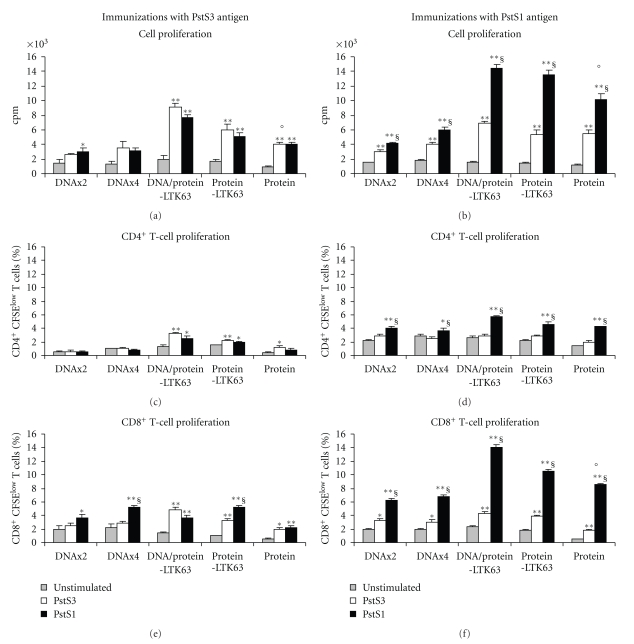
Cell proliferation in response to PstS1 or PstS3 proteins in spleen cells of immunized mice. Pooled spleen cells (3 mice/group) of mice vaccinated with PstS3 antigen (panels (a), (c), and (e)) or with PstS1 antigen (panels (b), (d), and (f)) were cultured at 4 × 10^5^ cells/well and re-stimulated *ex vivo* with PstS1 or PstS3 proteins (5 *μ*g/mL each) for 5 days before measuring thymidine incorporation in proliferating cells ((a) and (b)) or for 4 days before measuring CFSE dilution in replicating CD4^+^((c) and (d)) or CD8^+^ T cell populations ((e) and (f)). Data were combined from 3 independent experiments and are presented as mean. Error bars indicate SEM. The level of statistical significance for differences in each group of immunized mice was determined by a two-tailed Student's *t*-test (*P < .05; **P < .01 PstS1 or PstS3 stimulation versus unstimulated cells; ^§^between PstS1- and PstS3-induced responses in each group; °between antigen-specific responses observed in mice immunized with DNA or protein alone).

Spleen cells of immunized mice were re-stimulated *ex vivo* with PstS1 or PstS3 antigens and cell proliferation was measured by thymidine incorporation in total spleen cells at 5 days of culture or by CFSE dilution in CD4^+^ or CD8^+^ T cell populations after 4 days of culture (Figure 3).

Spleen cells of both PstS1 or PstS3 immunized mice proliferated in response to antigens with a greater contribution of CD8^+^ T cells rather than CD4^+^ T cells (Figures 3(c), 3(e), 3(d), and 3(f)). Proliferation was significantly higher by cells of mice immunized with protein rather than in cells of mice immunized with DNA especially in mice immunized with pstS1 antigen (Figures 3(a), 3(b), and 3(f)). Moreover in mice immunized with DNA only, CD8^+^ T cells were the cells mainly involved in proliferation (Figures 3(e) and 3(f)). While scarce or absent was the contribution of CD4^+^ T cells (Figures 3(c) and 3(d)). Administration of either PstS1 or PstS3 protein with LTK63 adjuvant did not significantly modify the antigen-specific memory response. Here again, some important differences between the two antigens were found. Mice immunized with PstS1 showed a greater cell proliferation compared to mice immunized with PstS3, while an anti-PstS1-specific CD4^+^ T cell proliferation, although weak, was found also in mice immunized with DNA only (Figure 3(d)). 

Cells of mice immunized with PstS1 proliferated also in response to PstS3 antigen stimulation but the effect was restricted to CD8^+^ T cell population, and the proliferation was significantly lower than that induced by PstS1 re-stimulation (Figure 3(f)). On the other hand, in cells of mice immunized with PstS3 antigen the magnitude of proliferation in response to both PstS1 and PstS3 antigen re-stimulation was similar and PstS1 was even better in re-activating CD8^+^ T cell proliferation in mice immunized four times with DNA or in the group immunized with protein and LTK63 (Figure 3(e)). Moreover, CD4^+^ T cells of some PstS3 immunizations (DNA/proteinLTK63 and protein LTK63-immunized mice) were able to proliferate in response to antigenic recall with PstS1 protein (Figure 3(c)).

These data suggested that PstS1 and PstS3 also differed in the capacity to induce and/or recall antigen-specific memory T cell proliferation.

### 3.3. PstS3 Antigen Was More Potent than PstS1 Antigen in Generating Memory IFN-*γ*-Producing Cells but Only PstS1 Protein Induced IL-17

**Figure 4:**
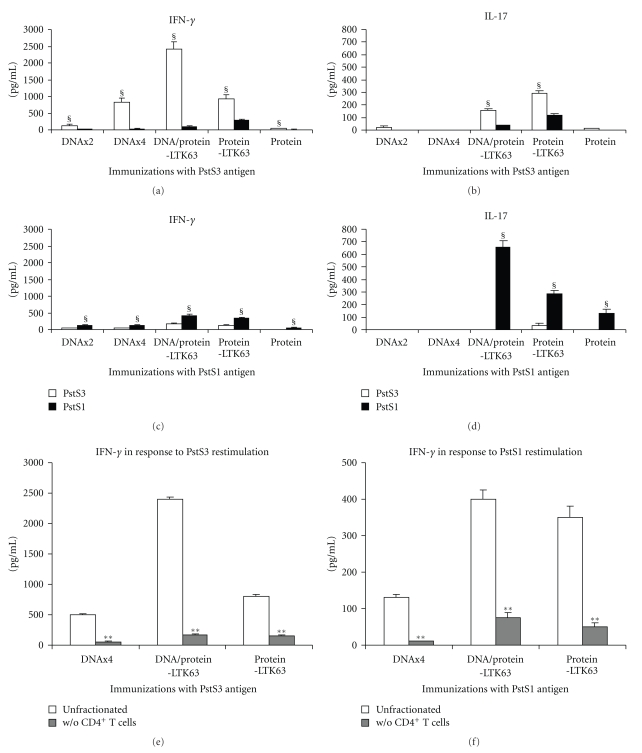
Cytokine production in response to PstS1 or PstS3 proteins in spleen cells of immunized mice. Pooled spleen cells (3 mice/group) of mice vaccinated with PstS3 antigen (panels (a) and (b)) or with PstS1 antigen (panels (c) and (d)) were cultured at 4 × 10^5^ cells/well and re-stimulated *ex vivo* with PstS1 or PstS3 proteins (5 *μ*g/mL each) for 4 days before measuring IFN-*γ* ((a) and (c)) or IL-17 ((b) and (d)) in the supernatant of culture by specific ELISA kit. Data were combined from 3 independent experiments and are presented as mean. Error bars indicate SEM. ^§^Statistical significant difference between PstS1- and PstS3-induced responses in each group of immunized mice (*P* < .05 or *P* < .01 determined by a two-tailed Student's *t*-test). In some experiments spleen cells of DNA x4-, DNA/protein-LTK63-, or protein/LTK63-immunized mice with both PstS3 (e) or PstS1 (f) antigens were depleted of CD4^+^ T cells by using magnetic beads as described in Materials and Methods. Undepleted cells and CD4^+^ T cell-depleted subset were re-stimulated with PstS1 or PstS3 protein (5 *μ*g/mL) for 4 days before testing the IFN-*γ* by a specific ELISA kit ((e) and (f)). Error bars indicate SEM. The level of statistical significance for differences among undepleted cells and CD4^+^ T cell-depleted subset in each group was determined by Student's *t*-test (*P < .05; **P < .01).

Spleen cells of immunized mice were re-stimulated in vitro with PstS1 or PstS3 antigen and after 4 days the supernatants of culture were analyzed to detect IFN-*γ* or IL-17 by ELISA. All kind of immunizations generated antigen-specific memory IFN-*γ*-secreting cells although the secretion was very low in mice immunized with protein alone or DNA only except in those animals receiving four administrations of DNA coding for *pstS3* (Figures 4(a) and 4(c)). Administration of protein with LTK63 greatly enhanced the IFN-*γ* response in the immunization with PstS3 antigen (Figure 4(a)) while the increase was modest, although still statistically significant, in PstS1 immunizations (Figure 4(c)). CD4^+^ T cells were mandatory for IFN-*γ* production as indicated by CD4^+^ T cell-depletion studies (Figures 4(e) and 4(f)). A weak cross-reactivity was observed with the related protein in all immunizations. In general, PstS3 antigen was a better inducer than PstS1 of memory IFN-*γ*-producing cells. 

The production of IL-17 was associated with the administration of PstS3 protein with adjuvant LTK63 (Figure 4(b)), while immunization with PstS1 protein even in the absence of adjuvant induced a specific Th17 response (Figure 4(d)). The production of IL-17 was higher in PstS1 immunizations compared to PstS3 immunizations. In PstS1-immunized mice the ratio of IL-17/IFN-*γ* was higher or around 1 indicating that the amount of the two cytokines released was similar (Figures 4(c) and 4(d)), while in PstS3 immunizations the ratio was much lower than 1 since the production of IFN-*γ* was always greater than IL-17 secretion (Figures 4(a) and 4(b)).

### 3.4. Neither PstS3 nor PstS1 Immunizations Reduced the Mycobacterial Load in the Lungs of Mice Challenged with *M. tuberculosis*

**Figure 5:**
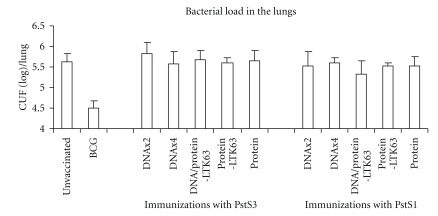
Effects of PstS1- or PstS3-immunizations on protection against *M. tuberculosis* challenge in mice. Mice (5 mice/group) were immunized with PstS1 or PstS3 antigens according to Figure 1, and challenged with *M. tuberculosis* H37Rv. Four weeks after infection, the bacteria in the lung were enumerated as described in Materials and Methods. Data are expressed as mean of the five individual mice ± SE. The level of statistical significance for differences between test groups and the control unvaccinated mice were determined by ANOVA test (*significant).

Four weeks after the last immunization, and concomitantly with the assessment of antigen-specific memory immune responses, groups of mice were challenged with virulent *M. tuberculosis* and the bacterial load in the lungs was measured 4 weeks after the challenge. In parallel experiments, protection induced by immunization with Ag85B under identical schedule was assessed (Table 1 [3, 4]). As shown in Figure 5, none of the vaccinations with PstS1 or PstS3 antigens was able to reduce the bacterial load in the lungs of infected mice. On the contrary, BCG vaccination (Figure 5 and Table 1) and all the several protocols of vaccination with the antigen Ag85, except only for immunization with Ag85B protein in absence of adjuvant, induced significant protection in the lungs of *M. tuberculosis*-infected mice (Table 1).

### 3.5. PstS1 and PstS3 Antigens in Contrast to Ag85B Were Not Recognized by IFN-*γ*-Secreting Spleen Cells of *M. tuberculosis*-Infected Mice: However PstS1 Stimulated IL-17 Production

**Figure 6:**
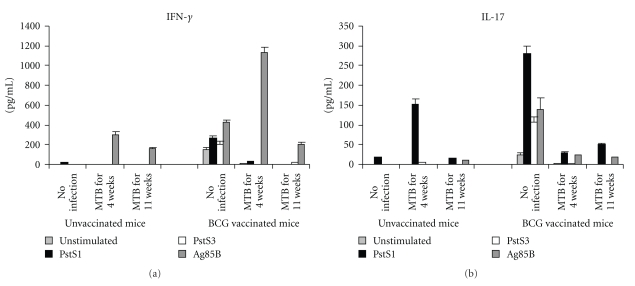
PstS1-specific IL-17—but not IFN-*γ*—secreting cells were generated during *M. tuberculosis* infection. Naïve unvaccinated or BCG-vaccinated mice (5 mice/group) were infected or not with *M. tuberculosis* for 4 or 11 weeks. Spleen cells recovered from mice were stimulated *ex vivo* (4 × 10^5^ cells/well) with PstS1, PstS3, or Ag85B proteins (5 *μ*g/mL each) for 4 days and culture cell supernatants assayed for IFN-*γ* (a) or IL-17 (b) by specific ELISA kits. Data were combined from 3 independent experiments and are presented as mean. Error bars indicate SEM.

To explain why the phosphate-binding protein immunizations were unsuccessful in protection against *M. tuberculosis* infection we investigated whether PstS1, or PstS3 specific immune reactions were raised during *M. tuberculosis* infection in unvaccinated or BCG-vaccinated mice. Therefore spleen cells of naïve unvaccinated or BCG-vaccinated mice recovered before *M. tuberculosis* infection or after 4 or 11 weeks from *M. tuberculosis* challenge were re-stimulated *ex vivo* with PstS1 or PstS3 proteins. Ag85B protein was also assayed as a protective comparator. The IFN-*γ*-secreting spleen cells of *M. tuberculosis*-infected mice responded to Ag85B protein, and the production of IFN-*γ* was greatly enhanced in mice vaccinated with BCG and infected for 4 weeks with *M. tuberculosis* (Figure 6). On the contrary, neither PstS3 nor PstS1 antigens were able to induce IFN-*γ* release by spleen cells of unvaccinated or BCG-vaccinated mice infected with *M. tuberculosis* for 4 or 11 weeks. Therefore, despite that PstS1 and especially PstS3 were good immunogens for activation of Th1 responses, the IFN-*γ* responses generated during early or late time points of *M. tuberculosis* infection (both in unvaccinated and BCG-vaccinated mice) were insensitive to these antigens. 

Completely different results were obtained for IL-17-secreting cells present in spleen cells of *M. tuberculosis*-infected mice. In fact, PstS1, but not PstS3, antigen stimulated Th17 response in *M. tuberculosis*-infected mice. An important production of IL-17 was found in spleen cells of unvaccinated mice infected with *M. tuberculosis* for 4 weeks and the PstS1-responding IL-17-secreting cells decreased with progression of *M. tuberculosis* infection. In mice vaccinated with BCG and infected with *M. tuberculosis* the PstS1-activated IL-17 response was similar at the two time points of infections. Moreover, PstS1 was a better inducer than Ag85B of Th17 response in *M. tuberculosis*-infected mice.

## 4. Discussion

The medical need of identifying new candidate antigens for vaccine development against TB led us to investigate the phosphate-binding transporter proteins of *M. tuberculosis*. In fact, these membrane lipoproteins have been reported to be virulence factors for *M. tuberculosis* [9], are expressed also on surface of BCG [8], and can induce specific immune response in patients with active TB [10, 12, 26]. Importantly, protection against *M. tuberculosis* challenge was reported in mice immunized with plasmid DNA coding for *pstS1* [14] or *pstS3* [13]. The aim of this paper was to reassess immunogenicity and protective capacity of the above proteins by using different protocols of vaccinations with PstS1 and PstS3 to diversify the antigen-specific immune response. Moreover, the data were compared with a well-known immunogenic and protective antigen, the Ag85B [3–5]. We noticed that both PstS1 and PstS3 are capable of inducing, to a various degree and magnitude, a number of immune responses which are usually considered to be relevant for immune protection, including activation of T cell-mediated immunity as exemplified by CD4^+^ and/or CD8^+^ T-cell proliferation and production of different amount of IFN-*γ* and/or IL-17. Although considered of scarce importance in protection against *M. tuberculosis* infection, antigen-specific humoral responses, especially in PstS1-immunized mice, were also induced. Nonetheless, neither PstS1- nor PstS3-immunized mice were protected from challenge with *M. tuberculosis,* at variance with BCG- or Ag85B-immunized mice. Lack of protection was observed regardless antigen formulation and expression, as protein or DNA or prime-boost immunization. Variations in methodology may explain why other research groups have found DNA-*pstS1 *[14] and DNA–*pstS3* [13] protective: Zhu et al. [14] challenged DNA-*pstS1*-immunized mice i.p. (not i.v.) with *M. tuberculosis* just 2 weeks (not 4 weeks) after the last booster. Tanghe et al. [13] challenged DNA-*pstS3*-immunized mice with a higher (10^6^ rather than 10^5^ mycobacterial cells) *M. tuberculosis* burden and this burden in the lungs was evaluated at 8 weeks (not 4 weeks).

The scarce/absent involvement of PstS3 and PstS1 antigen in the IFN-*γ* response, the essential arm of protective TB immunity [16–19], mounted in response to *M. tuberculosis* during natural infection may be the cause of inefficacy of vaccinations with these antigens. In fact, IFN-*γ*-secreting cells generated following *M. tuberculosis* infection, both in unvaccinated or BCG-vaccinated mice, did not respond to antigenic stimulation with PstS1 or PstS3 proteins while they were specific for the protective antigen Ag85B. The irrelevance of PstS3-specific IFN-*γ* immunity during *M. tuberculosis* infection was further confirmed by the fact that amplification of this response by priming/boosting regimen did not affect the replication of *M. tuberculosis* compared to unvaccinated or PstS3-vaccinated mice inducing low-IFN-*γ* response. 

Despite the fact that an s.c. immunization with PstS1 protein proved to be a nonprotective, it was recognized by IL-17-secreting cells generated during *M. tuberculosis* infection. The role of IL-17 in protection against *M. tuberculosis* infection is not fully elucidated. Infection of IL17^−/−^ mice with *M. tuberculosis* revealed that IL-17 was not essential to control the growth of *M. tuberculosis* during acute infection [19] suggesting that IFN-*γ*-secreting CD4^+^ and effector CD8^+^ T cells were sufficient to inhibit mycobacterial replication in the absence of IL-17. Recently, it has been reported that *M. bovis* BCG-specific Th17 cells confer partial protection against *M. tuberculosis* infection in the absence of IFN-*γ* [20], indicating that also Th17 cells per se, independently from IFN-*γ* response, may contribute to the early control of *M. tuberculosis* infection. However, the short-term protective effect provided by the IL-17-secreting cells occurred at the cost of increased tissue damage characterized by a marked neutrophil infiltrate [20]. In this context, one aspect of Th17 response is particular attracting in TB vaccination. IL-17 accelerates antigen-specific Th1 memory response in the lungs of vaccinated mice infected with *M. tuberculosis* [19]. One of the central improvements required in the development of effective TB vaccines is to shorten the delay in recruitment of antigen-specific Th1 cells in the lungs. Therefore, the immunogenic feature of PstS1 antigen to induce Th17 responses recognized during *M. tuberculosis* infection should be further investigated in TB vaccine development to study whether PstS1 given in combination with antigens inducing protective Th1 immunity could accelerate the expression of protective Th1 immunity in the lungs upon infection.

Another aspect of immunogenic features of PstS1 deserves to be further investigated. PstS1, even in the immunization schedules with protein alone, activated CD8^+^ T-cell proliferation even better than the CD4^+^ T-cell counterpart. Although the role of these PstS1-specific CD8^+^ T cells appears not uncoupled with a direct effect on protection, these cells might participate in the network of cellular regulation during infection. In fact, in addition to a recognized, direct role in protection against TB through their cytotoxic activity on *M. tuberculosis*-infected cells [27], CD8^+^ T cells specific for mycobacterial antigens can suppress IFN-*γ* production and proliferation by CD4^+^ T cells [28, 29].

LTK63 is a good adjuvant improving protection when used in vaccination with protective TB antigens [4, 5]. In vaccinations with antigen Ag85B, LTK63 improved protection against *M. tuberculosis* challenge by modulating memory IFN-*γ*-secreting CD4^+^ T cells with opposite effects. The INF-*γ* response was enhanced in unprimed mice vaccinated with Ag85B protein and adjuvant (Table 1) but reduced in mice primed with DNA and boosted with protein associated with the adjuvant [4]. In the latter case, coadministration of Ag85B protein with the adjuvant LTK63 reduced the generation of nonprotective Ag85B-specific IFN-*γ*-secreting cells, that led to a partial recovery in protection [4]. In fact, in DNA-primed mice boosted with adjuvant-free Ag85B protein, the expansion of an Ag85B-specific CD4^+^ T-cell subset secreting elevated IFN-*γ* amounts was associated with the loss of that protection conferred by immunization with DNA only [3]. Now we also report that LTK63 drives the commitment of memory antigen-specific T cells towards Th17 response, as observed not only in Ag85B but also in PstS1 and PstS3 immunizations. Considering that IFN-*γ* and IL-17 responses are negatively regulated by each other [30–32], the induction at the same time of both responses may help to prevent that one response, at the expense of the other, can expand without control causing inflammation-mediated lung damage during *M. tuberculosis* infection. In fact, the balance between protection and pathological consequences is the crux of TB pathogenesis. The ability to activate several mechanisms to contain uncontrolled expansion of Th1/Th17-mediated inflammatory process may be the reason of the efficacy of LTK63 adjuvant in TB vaccination.

## 5. Conclusion

We demonstrated that the *M. tuberculosis* phosphate-binding transporter lipoproteins PstS1 and PstS3 are excellent immunogens inducing CD8^+^ T-cell activation and both Th1 and Th17 immunity. These antigen-specific responses were not able, however, to contain *M. tuberculosis* replication in the lungs of infected mice even when amplified by administration of the protein with the adjuvant LTK63 or by the DNA priming/protein boosting regimen. The lack of protection might be ascribed to the scarce/absent immunogenicity/presentation of these antigens during natural infection. In fact, neither IFN-*γ*- nor IL-17-secreting cells specific for PstS3 were generated in spleen of unvaccinated or BCG-vaccinated mice infected with *M. tuberculosis*. Also PstS1 antigen, a weak inducer of Th1, did not react with IFN-*γ*-secreting cells generated during early or late infection with *M. tuberculosis*. However, although not determinant for protection, PstS1-specific IL-17-secreting cells were generated during *M. tuberculosis* infection both in unvaccinated and BCG-vaccinated mice. Although PstS1 is a nonprotective antigen “per se,” it could be utilized in TB vaccination, as modulator, in association with antigens making protective Th1 immunity. In fact, the PstS1 ability to drive Th17 immunity recognized upon *M. tuberculosis* infection could accelerate the recruitment of protective IFN-*γ* cells in the lungs of infected animals. To shorten the delay in recruitment of antigen-specific Th1 cells in the lungs is one of the central improvements required in the development of effective TB vaccines.

## Figures and Tables

**Table 1:** Effects of different immunizations with the mycobacterial antigen Ag85B on protection against *Mycobacterium tuberculosis* challenge and antigen-specific immunity in mice.

Immunizations^a^	IgG titer^b^	Cell proliferation (cpm)^c^	IFN-*γ* (pg/mL)^d^	IL-17 (pg/mL)^e^	−Δ CFU(log)/protection^f^	Reference^g^
DNA x2	800 ± 200	3500 ± 300	450 ± 30	0 ± 0	−0.7/yes	[3]
DNA x4	2000 ± 350	3937 ± 1064	559 ± 60	0 ± 0	−0.74/yes	[3]
DNA/protein-LTK63	43053 ± 4746	22037 ± 2695	6214 ± 168	320 ± 52	−0.32/yes	[4]
protein-LTK63	64507 ± 12388	16714 ± 326	3839 ± 150	420 ± 90	−0.31/yes	
protein	2800 ± 542	7579 ± 645	333 ± 100	10 ± 5	−0.02/no	[3]
BCG					−1.1/yes	[3, 4]

^a^mice (5 mice/group) were immunized with Ag85B according to Figure 1, and some mice challenged also with *M. tuberculosis* H37Rv.

^b^sera of immunized mice recovered 4 weeks from the last immunizations were analyzed by ELISA for the presence of anti-Ag85B Ab using a conjugated secondary Abs specific total IgG. Data are plotted as geometric mean ELISA titer ± SEM of 3 independent experiments.

^c^spleen cells of Ag85B-immunized mice were cultured at 4 × 10^5^ cells/well and re-stimulated *ex vivo* with Ag85B protein (5 *μ*g/mL) for 5 days before measuring thymidine incorporation in proliferating cells. Cell proliferation data are represented as mean cpm (subtracted from the cpm of unstimulated spleen cells) ± SEM of 3 independent experiments.

^d^spleen cells of Ag85B-immunized mice were cultured at 4 × 10^5^ cells/well and re-stimulated *ex vivo* with Ag85B protein (5 *μ*g/mL) for 4 days before measuring in cell culture supernatants IFN-*γ* by a specific ELISA kit. IFN-*γ* data are represented as mean (pg/mL) ± SEM of 3 independent experiments.

^e^spleen cells of Ag85B-immunized mice were cultured at 4 × 10^5^ cells/well and re-stimulated *ex vivo* with Ag85B protein (5 *μ*g/mL) for 4 days before measuring in cell culture supernatants IL-17 by a specific ELISA kit. IL-17 data are represented as mean (pg/mL) ± SEM of 3 independent experiments.

^f^After 4 weeks from *M. tuberculosis* challenge, the bacteria in the lung of infected mice were enumerated as described in Materials and Methods. Data are expressed as the Δ in CFU/lung (log) between vaccinated and unvaccinated control mice and it is indicated whether or not the vaccination was protective against *M. tuberculosis* challenge.

^g^Reference reporting some of the data.
